# Genomic Estimated Breeding Value of Milk Performance and Fertility Traits in the Russian Black-and-White Cattle Population

**DOI:** 10.32607/actanaturae.11648

**Published:** 2022

**Authors:** F. S. Sharko, A. Khatib, E. B. Prokhortchouk

**Affiliations:** Laboratory of vertebrate genomics and epigenomics, Federal Research Centre “Fundamentals of Biotechnology” of the Russian Academy of Sciences, Moscow, 119071 Russia; Laboratory I-Gene, ZAO “Genoanalytica”, Moscow, 119234 Russia; Department of biotechnology, faculty of Biology, Lomonosov Moscow State University, Moscow, 119234 Russia; Atomic Energy Commission of Syria (AECS), Department of Agriculture, Damascus, 6091 Syria

**Keywords:** GEBV, Russian Black-and-White cattle, genotyping, TD ssGBLUP-AM, test-day, milk performance, fertility

## Abstract

A breakthrough in cattle breeding was achieved with the incorporation of animal genomic data into breeding programs. The introduction of genomic selection has a major impact on traditional genetic assessment systems and animal genetic improvement programs. Since 2010, genomic selection has been officially introduced in the evaluation of the breeding and genetic potential of cattle in Europe, the U.S., Canada, and many other developed countries. The purpose of this study is to develop a system for a genomic evaluation of the breeding value of the domestic livestock of Black-and-White and Russian Holstein cattle based on 3 milk performance traits: daily milk yield (kg), daily milk fat (%), and daily milk protein content (%) and 6 fertility traits: age at first calving (AFC), calving interval (CI), calving to first insemination interval (CFI), interval between first and last insemination (IFL), days open (DO), and number of services (NS). We built a unified database of breeding animals from 523 breeding farms in the Russian Federation. The database included pedigree information on 2,551,529 cows and 69,131 bulls of the Russian Holstein and Black-and-White cattle breeds, as well as information on the milk performance of 1,597,426 cows with 4,771,366 completed lactations. The date of birth of the animals included in the database was between 1975 and 2017. Genotyping was performed in 672 animals using a BovineSNP50 v3 DNA Analysis BeadChip microarray (Illumina, USA). The genomic estimated breeding value (GEBV) was evaluated only for 644 animals (427 bulls and 217 cows) using the single-step genomic best linear unbiased prediction – animal model (ssGBLUP-AM). The mean genetic potential was +0.88 and +1.03 kg for the daily milk yield, -0.002% for the milk fat content, and –0.003 and 0.001% for the milk protein content in the cows and bulls, respectively. There was negative genetic progress in the fertility traits in the studied population between 1975 and 2017. The reliability of the estimated breeding value (EBV) for genotyped bulls ranged from 89 to 93% for the milk performance traits and 85 to 90% for the fertility traits, whereas the reliability of the genomic estimated breeding value (GEBV) varied 54 to 64% for the milk traits and 23 to 60% for the fertility traits. This result shows that it is possible to use the genomic estimated breeding value with rather high reliability to evaluate the domestic livestock of Russian Holstein and Black-and-White cattle breeds for fertility and milk performance traits. This system of genomic evaluation may help bring domestic breeding in line with modern competitive practices and estimate the breeding value of cattle at birth based on information on the animal’s genome.

## INTRODUCTION

One of the most challenging stages in the selection of farm animals is the assessment of their breeding value. To evaluate the breeding value, it is necessary to compare and analyze the breeding characteristics of the animals being evaluated, their closest relatives, offspring, and ancestors. At the initial stages of the development of livestock breeding, the breeding value was assessed by phenotypic indicators: in particular, milk performance indicators were used in dairy cattle breeding [1, 2]. However, more efficient methods based on molecular genetic markers have been developed in the last decade in order to assess the breeding value. Significant progress has been achieved thanks to success in deciphering the genome of the main agricultural animals (cattle, pigs, and sheep) [3], as well as the use of statistical analysis, in particular the best linear, unbiased prediction (BLUP) method. Calculation of the breeding value using the BLUP method makes it possible to exclude the influence of non-genetic factors on the variability of the selected traits in a population, as well as to identify and evaluate the genetic component with a high degree of reliability [4]. The use of molecular genetic markers improves reliability in the assessment of the breeding value of young animals, reduces the generation interval, and expands the capabilities of intensive selection. In addition, the use of genomic assessment leads to an increase in the rate of genetic improvement of economically useful traits in cows and to a decrease in material and technical costs in assessing the genetic potential of sires [5, 6]. Genomic assessment is of particular importance for health and fertility indicators, because the reliability of a genomic assessment of the breeding value is only slightly inferior to the reliability of these indicators for the quality of offspring. To date, there has been no significant genetic progress in the assessment of fertility traits, because, for a long time, many of these traits have not received the appropriate level of attention in breeding programs [7]. The reliability of the genomic estimated breeding value of young animals depends on the reliability of the assessment of the animals included in the reference population – a population of sires with a highly reliable assessment of the offspring and available genomic information [8]. Because obtaining information about the genome is a standardized and proven technology, the reliability of the genomic estimated breeding value largely depends on the reliability of the assessment of the animals included in the reference population based on the offspring. In practice, assessment of the breeding value for milk performance traits by offspring is based on the use of a 305-day lactation yield [9]. The 305-day milk yield is calculated using daily measurements of milk production and the percentage of milk fat and protein for a month. These measurements are called test days [10]. The use of the 305-day milk yield to evaluate the estimated breeding value not only has some advantages, but also a number of disadvantages. First, the procedure for calculating the milk yield [11] is based on plotting the lactation curve using the test day results with fixed parameters, which leads to an underestimation of milk performance during the first months of lactation and to its overestimation during the last months of lactation. These wrinkles may lead to an incorrect calculation of the 305-day milk yield and a decrease in the reliability of the estimated breeding value based on these source data and, therefore, to a decrease in the reliability of the genomic estimated breeding value. Second, when using the 305-day milk yield in linear and non-linear mathematical models as a fixed factor influencing the variability of this value, an averaged effect of the environment for this lactation (herd–year–calving season effect) is used, which means that the effect is constant throughout lactation [12]. In practice, this effect may vary greatly from one lactation day to the next [13]. Ignoring the variability of the environmental effect on the daily milk yield leads to incorrect calculation of genetic and paratypic parameters upon assessment of the breeding value and also introduces an error in the assessment of the breeding value by the offspring and genome. Using the daily milk yield results directly in the generation of mathematical models for the assessment of the breeding value solves all the problems mentioned above [14]. These mathematical models are called test day models or TD models [15]. The purpose of this study was to develop a system for genomic evaluation of the breeding value of the domestic stock of Holstein and Black-and-White cattle using the TD ssGBLUP-AM method based on a set of milk performance traits (daily milk yield (kg), milk fat (%), milk protein (%)) and the ssGBLUP-AM method for fertility traits: age at first calving (AFC, days), calving interval (CI, days), calving to first insemination interval (CFI, days), interval between first and last insemination (IFL, days), days open (DO, days), and number of services (NS).

## EXPERIMENTAL

**Database of breeding animals**

We developed a unified database on the phenotypic indicators of the studied traits and the pedigree of animals from 12 regions of the Russian Federation. To develop the unified database, we used primary databases about animals from 523 farms included in the register of breeding organizations of the Ministry of Agriculture of the Russian Federation. Primary raw data were obtained as databases generated using the SELEX software package [16], which is related to RDBMS Firebird 2.5. Operating the databases and unloading the necessary information were performed using the Python 2.7 programming language and FDB package. Information on fertility and milk performance indicators and the pedigree of each animal with completed lactation from 523 local databases was uploaded. Information about milk performance for each animal included information about TD (the day of collection of animal milk performance at the control milking day) for each lactation: daily milk yield, daily fat percentage, and daily protein percentage. Information for the database of phenotypic data on fertility traits included information about the date of calving for each animal, age at calving, and date and number of services. Also, we uploaded all primary information about the pedigree of all animals with known productivity and information about all known generations of ancestors on the paternal and maternal lines.

**System for assessing the reliability of the phenotypic data of the breeding animals included in the created database**

An analysis of the unified database of the breeding animals revealed that the primary data contain numerous errors and inaccuracies. This prevented the use of these data in further research. To correct the situation, a unique multi-stage system for checking the reliability of milk performance data was developed. It included six main stages: checking data for critical values, checking the duration of pregnancy, checking the variability of milk performance data within each farm, checking the number of test days in lactation, and analyzing the reliability of milk performance data within each lactation. All lactations included in the created unified database were checked sequentially at each stage. Lactations that did not pass quality control were removed from further analysis.

First, milk performance data whose values were less than or equal to 0 were removed from the database. Next, the milk performance data were checked for falling into the interval (µ– 3σ, µ+ 3σ), and those that did not fall in the interval were removed. It should be noted that not only daily milk yield values, but also data on milk fat and milk protein content were deleted, regardless of whether they passed the test or not.

At the next stage, the duration of pregnancy for each lactation was also checked using the three-sigma rule [17]. Erroneous non-positive values were preliminarily removed. As a result, lactations that corresponded to a pregnancy duration of 268 to 317 days were tested. Lactations whose duration of pre-pregnancy did not fall within the confidence interval were excluded from further analysis.

At the third checking stage, the variability of the traits within the herd at each farm was controlled to exclude data obtained by copying one-shot values. This checking eliminated trait values at each farm from further analysis if the same values were found in the data of the farm for each control dairy day, week, or month.

The next step in checking milk performance data was to check the number and quality of the test days in each lactation. According to the accepted rules for assessing the milk performance of cows [18], the data were checked for meeting the following conditions:

1) there should be data on at least three test days in lactation;

2) there should be no more than 70 days between the calving date and the first TD date;

3) there should be no more than 70 days between adjacent TDs.

Lactations that did not fit these rules were removed. It should be noted that if the "daily milk yield in kg" data were deleted, then the entire lactation was deleted.

At the next checking stage, a lactation curve was built using internationally recognized methods [19, 20] for each lactation for which the information on daily milk yields passed the previous checking stages. For each plotted lactation curve, the mean absolute approximation error (MAE) was calculated for each trait. The results obtained for each trait form a normally distributed sample of values. On the basis of the analysis of the calculated mean absolute approximation errors for each lactation, lactations that had too large an approximation error (were not within the interval (0, µ+ 3σ)) were excluded from further calculations.

Primary data on fertility traits were checked for each trait separately. Regarding the age at first calving, data whose values did not fall into an interval of 18–30 months were deleted. Regarding calving interval data, the database included only those lactations that corresponded to a calving interval of 300 to 600 days. Also, the database included data whose values ranged from 25 to 360 days for the calving interval trait – first insemination (CFI) and 25 to 500 days for first–last insemination (IFL) and days open (DO) traits. The reliability of the data on the number of services (NS) was checked for compliance with the condition that this value should not exceed 10 inseminations per lactation.

**System for assessing the reliability of information on the pedigree of the breeding animals**

Information about the pedigree of all animals whose lactation data were deleted during checking of the reliability of fertility and milk performance data was deleted from the unified database. A primary analysis showed that the quality of the data on the pedigree of the animals precluded their further analysis because of a large number of duplicates, errors, and inaccuracies.

At the first stage of correcting the data on the pedigree of the animals, a unique algorithm for correcting loops in the existing primary database was developed. The main idea behind the algorithm is to assign a generation number to an animal and analyze its changes. Initially, each animal in the kinship table has a value of 1. If sequential passing through the table encounters descendants of an animal, then the number of the appropriate generation is increased by one. If the offspring of an animal has a higher generation number, then the generation number of the animal should be proportionally increased. The algorithm operates until the numbers of animal generations stop changing. Accordingly, the animal with the highest generation number is the ancestor. If there are errors in the data of some animal and there are cycles, its generation number will not stop increasing. Animals with this anomaly were removed from the pedigree database. The developed algorithm enabled the removal of erroneous data of this kind.

The next step in adjusting the constituted kinship database was the formation of a combined database on the pedigree of the animals using a reference database. This stage included integrating animals from the created database into the CDCB (Council of Dairy Cattle Breeding, USA) [21], which is publicly available and is the most complete database of dairy breeds in the world. Information on the animal pedigree obtained from this database was considered as the reference. Further, data on the pedigree of animals obtained from Russian and foreign sources were used to generate two genealogical trees and perform a search for matches at the tops of these trees. Search conditions were matching of gender + part of the number + date of birth or matching of gender + number with a length of more than 7 digits. If the vertices coincided, all records about the ancestors of the animal, which were obtained from Russian sources, were replaced with reference ones. This, among other things, satisfied the lack of information in the databases and the combined branches of the genealogical tree built based on Russian data, which would never have crossed without a foreign database.

After developing the combined database on the pedigree of the animals, grouping of duplicates of the same ancestors of the animals with completed lactation was performed. First, records that had not been replaced at the previous stage were pooled according to the coincidence of nickname + date of birth, or inventory number + nickname, or inventory number + date of birth. Each group of records was assigned a unique number in chronological order. Erroneous data were deleted if two or more unique numbers were assigned to parents (father or mother) in one group of records. Further, the data were grouped with allowance for sibling relationships (match of father or mother + match of any personal data (nicknames, numbers or dates of birth)).

We also tested a method for the recovery of some missing information in the relationship matrix by iteratively estimating the matrix R (covariance matrix of the residual error e) for the AM model. We applied the EM algorithm [22], an algorithm used in mathematical statistics to find maximum likelihood estimates for the parameters of probabilistic models when the model depended on some hidden variables. First, latent variables are estimated by the current approximation of parameters and, then, the parameter estimate that maximizes the likelihood of the latent variable is estimated and repeated until it converges to the maximum likelihood. As a first approximation, we assumed that the matrix R was diagonal. By solving the AM model with it, we obtained an estimate of the internal parameters (β and u) of the model; in the next iteration, we found the next approximation of the estimate of the matrix R using the same AM model, thereby improving the accuracy of our AM model estimate.

**Animal genotyping**

We genotyped 672 animals. DNA was isolated from blood and skin notches according to the standard QIAamp® DNA Investigator protocol. Samples containing 4 µl of a DNA solution with a concentration of 50 ng/µl were genotyped using a BovineSNP50 v3 DNA Analysis BeadChip microarray (Illumina, USA), according to the manufacturer’s instruction. Only genotypes with a call rate > 90% were used to develop a system for genomic evaluation of the breeding value. All SNP markers with a minor allele frequency of less than 5% were excluded from the analysis.

**Determination of breed by Principal Component Analysis (PCA)**

Using the PCA method, we were able to tentatively identify the breeds of the animals, information on which was not available in our database. In this method, we used the genotypes of 672 animals of various breeds and the plink program.

**Fig. 1:**
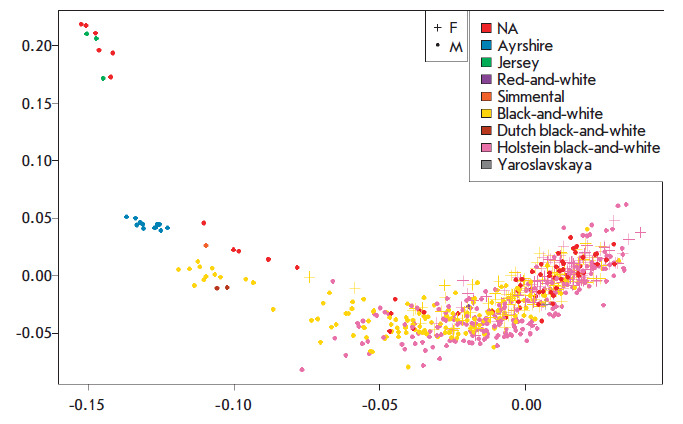
Principal component analysis PC1 and PC2 (PCA) for the genotyped animals

Thus, Fig. 1 shows a clear separation of Jersey and Ayrshire animals and a large cluster of animals from the Black-and-White family. The method enabled the identification of 644 animals (427 sires and 217 cows) belonging to the Holstein (392) and Black-and-White (252) breeds, which were subsequently used to assess the breeding value.

**Estimation of the breeding value and genetic parameters of the Black-and-White animal population**

The breeding value of the animals was assessed using the TD ssGBLUP AM method [23, 24] for milk performance traits and the ssGBLUP AM method [25] for fertility traits. The following fixed models were created:

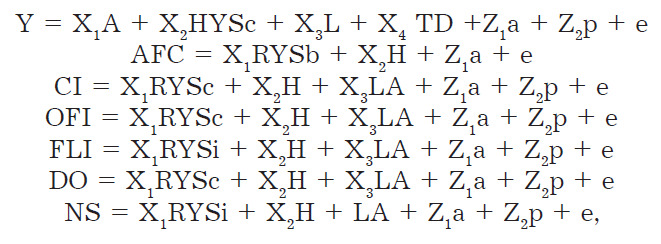



where Y is a vector of the milk performance traits (milk yield (kg), fat content (%), milk fat yield (kg); protein content (%), and milk protein yield (kg)); AFC is the vector of the age at first calving trait (days); CI is the vector of the calving interval trait (days); CFI is the vector of the calving to first insemination interval trait (days); IFL is the vector of the interval between first and last insemination trait (days); DO is the vector of the days open trait (days); NS is the vector of the number of services trait; A is the fixed effect vector of animal age; HYSc is the fixed effect vector of farm–year–calving season; RYSb is the fixed effect vector of region–year–season of birth; L is the fixed effect vector of lactation number; H is the fixed effect vector of farm; TD is the fixed effect vector of control milk day; RYSc is the fixed effect vector of region– year–calving season; RYSi is the fixed effect vector of region–year–season of insemination; LA is the fixed effect vector of lactation–animal age; a is the vector of animal randomized additive effects; p is the vector of randomized environmental effects; e is the residual effect vector; and X_1_, X_2_, X_3_, Z_1_, and Z_2_ are unit diagonal matrices relating the vector of observation to the fixed and random effect vectors.

The genetic parameters (heritability and repeatability coefficients) were calculated according to the following formulas [26]:

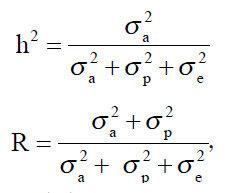



where h^2^ is the heritability coefficient; R is the repeatability factor; σ_a_^2^ is the additive genetic variance; σ_p_^2^ is the environment variance; and σ_e_^2^ is the residual effect variance.

The reliability of the estimated breeding value was calculated using the following formula [27]:

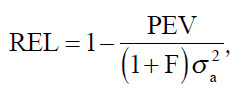



where REL is the reliability of the estimated breeding value, PEV is the predicted error variance; F is the inbreeding coefficient and σa2 is the additive genetic variance.

## RESULTS

**Characterization of the database of breeding animals of the Russian Black-and-White cattle population**

**Fig. 2:**
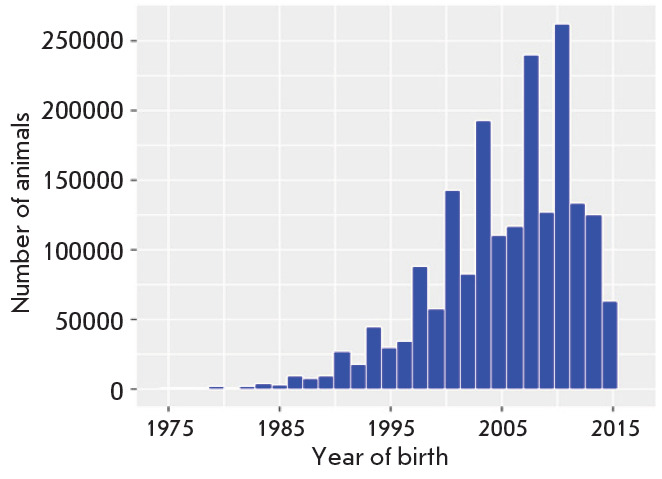
Distribution of animals in the database by date of birth

**Fig. 3:**
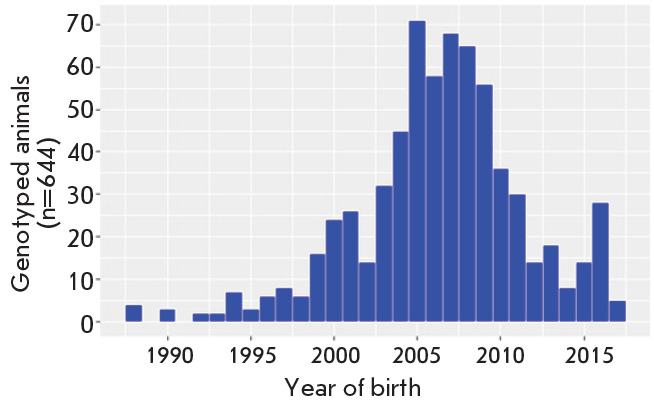
Distribution of the genotyped animals by date of birth

The developed system was used to form a unique, consolidated database on the pedigree of breeding animals on the paternal and maternal lines, which included information on 69,131 bulls and 251,529 cows of the Black-and-White dairy breed. The developed system enables a combination of heterogeneous information on the pedigree of dairy breeding animals from 523 farms in the Russian Federation. The birth dates of the animals according to lactations included in the database were distributed between 1975 and 2017; the mean number of test days per lactation was 9. The distribution of the animals in the developed database and the distribution of genotyped animals by date of birth are shown in Fig. 2 and Fig. 3.

After a test of the system for checking phenotypic data and the data on the pedigree of the animals, the final database included information on 1,597,426 cows with 4,771,366 completed lactations. There are data on the daily milk yield, milk fat, and milk protein in 1,047,224, 1,033,839, and 1,046,148 animals, respectively. The number of test day records for the daily milk yield, milk fat, and milk protein content was 29,735,417, 26,393,276, and 26,955,476, respectively. The kinship table for three milk performance traits contained information on 1,983,031 animals, of which 51,810 were sires. The mean performance value of the entire livestock was 20.9 ± 8.433 kg for the daily milk yield, 3.90 ± 0.46% for the milk fat content, and 3.18 ± 0.24% for the milk protein content. The mean age at first calving was 836.06 ± 117.32 days. For other fertility indicators, the mean value was: 401.79 ± 67.098 days for the calving interval, 90.713 ± 53.425 days for the calving–first insemination interval, 41.685 ± 79.243 days for the interval between first–last insemination, 140.18 ± 89.805 days for days open, and 1.80 ± 1.39 for the number of services (Table 1).

**Table 1:** Indicators of breeding animals from the Russian Holstein and Black-and-White cattle populations

Trait	Number of animals	Number of records	Number of animals in kinship table	Number of bulls	Min	Max	Mean	Standard deviation
Daily yield, kg	1,047,224	29,735,417	1,983,031	51,810	0.2	46.211	20.90	8.43
Milk fat, %	1,033,839	26,393,276	1,983,031	51,810	2.38	5.47	3.90	0.46
Milk protein, %	1,046,148	26,955,476	1,983,031	51,810	2.31	4.08	3.18	0.24
AFC, days	937,175	937,175	1,434,321	49,644	540	1230	836.06	117.32
CI, days	763,773	2,026,259	1,247,553	46,371	300	600	401.79	67.1
CFI, days	904,999	2,535,158	1,409,240	49,111	25	360	90.713	53.43
IFL, days	787,536	3,174,412	1,214,206	47,352	0	720	41.685	79.24
DO, days	898,131	2,539,399	1,400,007	48,964	25	500	140.18	89.81
NS	959,501	3,575,124	1,447,815	49,781	1	10	1.80	1.39

**Evaluation of the genetic parameters of breeding traits in the Holstein and Black-and-White cattle population**

To assess the breeding value of animals in a cattle population, it is necessary to determine the parameters of breeding traits in the animals in the population. The following genetic parameters of the Russian Holstein and Black-and-White cattle populations were evaluated: phenotypic variance, genetic variance, environment variance, residual variance, repeatability coefficient, and heritability coefficient. To calculate the dispersion components, the AIREMLF90 module was used, which, in turn, is based on the AI-REML (Average Information-Residual Maximal Likelihood) algorithm. The calculated genetic parameters are presented in Table 2.

**Table 2:** Calculation of the genetic variance (σ2 a), environment variance (σ2 p), residual variance (σ2 e), repeatability coefficient (R), and heritability coefficient (h2)

Trait	σ^2^_a_	σ^2^_p_	σ^2^_e_	h^2^	R
Daily milk yield, kg	4.644 ± 0.783	5.278 ± 0.545	13.536 ± 0.112	0.20	0.427
Milk fat, %	0.108 ± 0.189	0.109 ± 0.130	0.127 ± 0.610	0.31	0.631
Milk protein, %	0.221 ± 0.431	0.261 ± 0.302	0.364 ± 0.172	0.26	0.569
AFC, days	2,025 ± 24.12	–	7,515 ± 19.09	0.21	–
CI, days	215.98 ± 4.896	334.3 ± 4.762	3,736.6 ± 4.646	0.05	0.13
CFI, days	232.02 ± 3.172	147.42 ± 2.569	2,187.5 ± 2.375	0.09	0.15
IFL, days	296.58 ± 5.141	438.19 ± 4.523	4,861.7 ± 4.462	0.05	0.13
DO, days	505.30 ± 9.534	1,070.8 ± 8.925	6,183.1 ± 6.994	0.07	0.2
NS	0.961 ± 0.423	0.522 ± 0.341	0.731 ± 0.254	0.11	0.19

The calculation of variance components shows that the variability of the fertility and milk performance traits in the Holstein and Black-and-White cattle populations in Russia is quite high, which makes targeted breeding for these traits quite effective. The heritability coefficient was 0.20 for the daily milk yield, 0.31 for the milk fat content, and 0.26 for the milk protein content. For all fertility traits, except for AFC, the heritability coefficient was low; < 0.11. This indicates a low genotypic diversity of the animal population and a high impact of environmental conditions on the variability of these traits.

**Evaluation of the breeding value of cows and sires of Holstein and Black-and-White breeds**

We calculated the genomic breeding value of all the animals born between 1975 and 2017 and represented in the developed database. The estimated breeding value (EBV) was calculated using the ssGBLUP-AM method. This method enables the inclusion of information about the phenotype and genotype of the animals and the pedigree of the animals into a single model. The BLUPF90 software [27] was used at all steps of breeding value assessment. The result of the breeding value evaluation is provided in Table 3.

**Table 3:** Evaluation of the breeding value of cows and sires for the main breeding traits of fertility and milk performance in the Holstein and Black-and-White cattle populations

Trait	EBV (cows)				EBV (bulls)			
	Min	Max	Mean	Reliability (mean)	Min	Max	Mean	Reliability (mean)
Daily milk yield, kg	–11.23	13.98	0.88	0.38	–12.05	15.07	1.03	0.33
Milk fat, %	–0.55	0.69	-0.002	0.39	–0.97	0.73	–0.002	0.34
Milk protein, %	–0.22	0.31	–0.003	0.37	–0.18	0.30	0.001	0.32
AFC, days	–142.66	170.45	–11.35	0.35	–199.83	198.35	–10.67	0.32
CI, days	–34.37	49.54	2.76	0.28	–36.68	49.88	3.07	0.26
CFI, days	–51.45	56.24	–2.02	0.33	–66.5	73.9	–0.73	0.30
IFL, days	–40.38	82.07	5.93	0.30	–48.52	94.69	5.85	0.27
DO, days	–53.94	72.18	3.25	0.29	–68.83	106.07	4.14	0.27
NS	–1.03	2.18	0.14	0.23	–1.08	1.66	0.05	0.21

The mean genetic potential was 0.88 kg in cows and 1.03 kg in bulls for the daily milk yield, –0.002% for the milk fat content, and –0.003 and 0.001% for the milk protein content in cows and the progeny of bulls, respectively. It should be noted that the mean assessment values for each trait are close to zero, and that the distribution of the animals relative to this value is almost symmetrical (1 : 1): i.e., 50% of the animals have positive values and the other 50% have negative values. The genetic trend for the main breeding traits of fertility and milk performance in the Black-and-White breed population is built using the mean calculated breeding value of the animals by year of birth and is shown in Fig. 1 (Appendix).

A significant increase in the milk yield (4.4 kg/day) was observed between 1975 and 2017, while a decrease in the milk protein content was noted between 1975 and 2002. Then, between 2002 and 2017, the mean breeding value of the animals increased from –0.006 to 0.002%. After 2010, the genetic trend in the fat content shows a significant drop from –0.005 to –0.03%. A decrease in all fertility indicators, except for the age at first calving, occurred between 1975 and 2017.

One of the factors affecting the accuracy of the breeding value estimate is the level of trait heritability. The higher the heritability, the higher the estimate accuracy. In our study, the EBV accuracy for three milk performance traits and the AFC fertility trait is higher than the EBV accuracy for other fertility traits (CI, CFI, IFL, DO, and NS). However, the heritability coefficient varied from 0.20 to 0.31 for the AFC and milk traits and from 0.05 to 0.11 for other fertility traits.

**Assessment of the effectiveness of the system for genomic evaluation of dairy cattle**

The reliability of the genomic estimated breeding value was evaluated using cross-validation. Genotyped animals were randomly divided into 11 equal groups. Ten groups were used in turn to calculate the model. The remaining 11th group was a test group: data on the descendants of the animals in this group were deleted, and the breeding value was calculated only based on the genome. Then, the breeding value of the animals was compared to their breeding value using phenotypic data. The degree of correlation between the breeding value of the genotyped animals, which was calculated by offspring (EBV), and their breeding value calculated by genotype (GEBV) served as a criterion for the reliability of the genomic estimated breeding value. The result of our assessment of the reliability of the genomic prediction is presented in Table 4.

**Table 4:** Calculation of the reliability of the genomic-estimated breeding value for the main breeding traits of fertility and milk performance in the Black-and-White cattle population

Trait	Genotyped cows (n = 217)			Genotyped bulls (n = 427)		
	Number of offspring (mean)	Reliability of EBV	Reliability of GEBV*	Number of offspring (mean)	Reliability of EBV	Reliability of GEBV*
Daily milk yield, kg	1.02	0.59	0.98	583.2	0.93	0.65
Milk fat, %	1.02	0.59	0.97	583.2	0.92	0.54
Milk protein, %	1.02	0.57	0.97	583.2	0.89	0.54
AFC, days	0.08	0.21	0.82	358.2	0.89	0.24
CI, days	0.05	0.15	0.87	285.1	0.87	0.60
CFI, days	0.08	0.21	0.93	347.	0.87	0.45
IFL, days	0.08	0.20	0.54	219.4	0.86	0.26
DO, days	0.08	0.20	0.93	345.5	0.90	0.56
NS	0.09	0.18	0.51	359.1	0.85	0.23

^*^Reliability of the estimate compared to the estimate for offspring (square of the rank correlation coefficient).

The accuracy in the assessment of the breeding value by offspring (EBV) was calculated based on the variational components and genetic variability of the traits using the REML method, and the accuracy of GEBV was calculated as the square of the rank correlation coefficient between the EBV and GEBV values. It is worth noting that bulls have significantly more offspring than cows. In this study, the mean number of offspring in the genotyped bulls ranged from 219.4 for IFL to 583.2 for milk performance traits. In the genotyped cows, the mean number of offspring did not exceed 1.02 for all the studied traits, while the reliability of EBV mainly depended on the number of offspring. As shown in Table 4, the reliability of EBV in the genotyped cows is less than that in the bulls. In the genotyped bulls, a high accuracy of EBV (> 85%) is observed for all fertility and milk performance traits; in the genotyped cows, the reliability of EBV ranges from 0.18 for NS to 0.59 for the milk yield and milk fat content.

The correlation of EBV and GEBV (reliability of GEBV) exceeded 80% in the genotyped cows for most of the studied traits and reached 98% for the daily milk yield.

When calculating GEBV, offspring data of genotyped animals were removed and the breeding value was assessed only by genotype. Genotyped cows have few offspring, so removal of offspring from the ssGBLUP model does not significantly affect the EBV values of the animals and, thus, there is a high correlation between the EBV and GEBV values. Therefore, unlike sires, the reliability of GEBV in genotyped cows may not reflect the effectiveness of the genomic scoring system.

In genotyped bulls, mean values of GEBV reliability were found for three milk performance traits. This result indicates the possibility of assessing the breeding value of the Black-and-White cattle population by genotype with a reliability of up to 65% for the daily milk yield and up to 54% for the milk fat and protein content. A rather high accuracy of GEBV was found for the CI, DO, and CFI traits: 60, 54, and 45%, respectively. The minimum GEBV accuracy was obtained for AFC (24%), IFL (26%), and NS (23%) traits.

The advent of genomic selection has reduced the requirements on traditional approaches to choosing candidates for selection when many phenotypic traits of all close relatives of candidates should be determined. Genomic selection opens up the opportunity for selecting traits that are difficult or expensive to measure, such as fertility. This approach will be developed through new genomic studies (based on genomics, transcriptomics, and proteomics) aimed at identifying the genes and pathways that control fertility in cattle and will improve phenotyping for reproductive function.

**Fig. 4:**
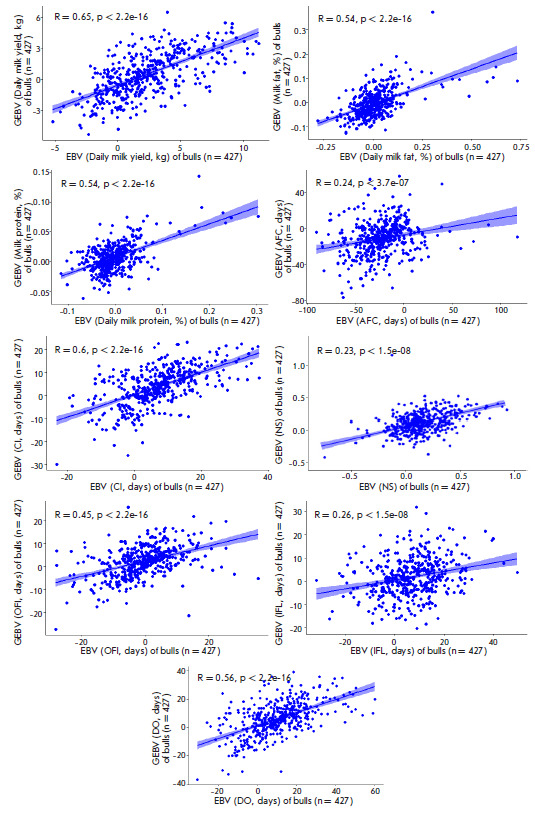
Correlation between the estimated breeding values (EBV) of bulls and their genomic estimated breeding values (GEBV) for the fertility and milk performance traits

**Fig. 5:**
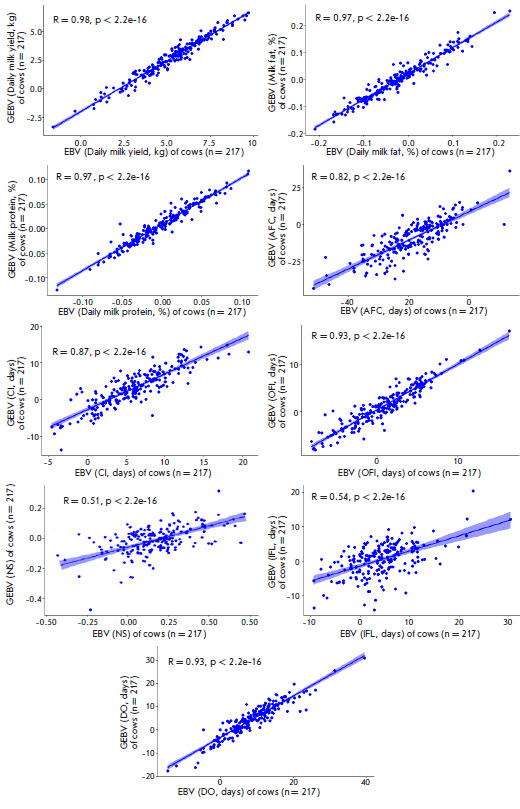
Correlation between the estimated breeding values of the genotyped cows (EBV) and their genomic-estimated breeding values (GEBV) for the fertility and milk performance traits

The result of an evaluation of the reliability of the genomic prediction in genotyped bulls and cows is also shown in Fig. 4 and Fig. 5.

## DISCUSSION

The breeding results confirmed that prediction of the cattle breeding value using genomic information is more accurate than kinship, alone [28, 29, 30]. In this study, the genomic estimated breeding value in the Russian Holstein and Black-and-White cattle populations was determined for the first time in Russia based on fertility traits. The reliability of the genomic estimated breeding value was 65% for the daily milk yield and 54% for the milk fat and protein content. The reliability of GEBV for fertility traits amounted to 60% (CI), 54% (DO), 45% (CFI), 24% (AFC), 26% (IFL), and 23% (NS). These values are slightly higher than those in Nordic Red dairy cattle (from 0.22 to 0.31%) for three fertility traits [31]. A similar result (28.9% reliability) was obtained by Su et al., who assessed the breeding value in Danish Jersey using a small reference population (1,250 Danish bulls) [32].

In addition, we used the TD ssGBLUP-AM test day model to assess the breeding value of cattle for milk traits. Currently, this model is used to officially assess the cattle breeding value in many countries: e.g., Nordic Red dairy cattle (RDC) [33]. The official RDC assessment data for March 2012 were obtained from a genetic assessment of Nordic cattle (NAV). To assess the breeding value of RDC, 3,538,966 cows were selected from 95.6 million records of test days and the total number of animals in the RDC pedigree was 477,468 (Table 5). Comparison of the results of our earlier study of the Holstein Dairy breed shows that despite a 2.5-fold difference in the size of the statistical sample, assessment of the breeding value of the Holstein and Black-and-White breeds by the TD ssGBLUP-AM method has a rather high prediction reliability (about 65%).

**Table 5:** Comparison of the Black-and-White breed with various cattle breeds in the world

Breed	Number of cows		
	Milk yield, kg	Milk fat	Milk protein
Black-and-white	1,047,224	1,033,839	1,046,148
Nordic RDC	3,538,966	3,538,966	3,538,966
Holstein (Canada)	5,976,711	5,976,711	5,976,711
Ayrshire (Canada)	221,533	221,533	221,533
Jersey (Canada)	185,737	185,737	185,737
Portuguese Holstein	578,552	–	–
German Holstein	48,977	–	–
	Number of test days, million		
	Milk yield, kg	Milk fat	Milk protein
Black-and-white	29.7	26.4	27
Nordic RDC	95.6	95.6	95.6
Holstein (Canada)	72.4	72.4	72.4
Ayrshire (Canada)	2.4	2.4	2.4
Jersey (Canada)	1.7	1.7	1.7
Portuguese Holstein	11.4	–	–
German Holstein	0.106		
	Reliability of GEBV, %		
	Milk yield, kg	Milk fat	Milk protein
Black-and-white	65	54	54
Nordic RDC	40	50	40
Holstein (Canada)	65	58	67
Ayrshire (Canada)	39	43	54
Jersey (Canada)	58	62	68
Portuguese Holstein	52–72	–	–
German Holstein	81–88	–	–

The calculated genomic prediction reliability of the breeding value is comparable with the estimated breeding value of Portuguese Holstein cows [34]. The mean reliability of the genomic-estimated breeding value of Portuguese Holstein bulls was 52% in young bulls and 72% in bulls with data on the performance of their daughters.

The test day model is likewise used for a genomic estimate of the breeding value in three dairy cattle populations in Canada (Holstein, Ayrshire, and Jersey). The prediction reliability of the breeding value for the milk yield is 65, 39, and 58% for Holstein, Ayrshire, and Jersey breeds, respectively [35]. In a study by Bohlouli et al., 11.4 million test day records were used to estimate the breeding value of 48,977 Holstein cows in Germany. The reliability of the evaluation reached 88% [36].

## CONCLUSION

In this study, despite a small number of genotyped sires in the reference population, an acceptable level of reliability in the genomic assessment of the cattle breeding value was achieved. Reliability may be improved by increasing the number of genotyped animals in the reference population. We have shown that there is a possibility to use the genomic-estimated breeding value in the domestic population of Holstein and Black-and-White cattle according to various fertility and milk performance traits. This system will take domestic breeding to a modern, competitive level and help evaluate the cattle breeding value at birth based on information about the animal’s genome.
